# CREATION OF A FAMILY CAREGIVER NEGOTIATION TRAINING PROGRAM USING ARTIFICIAL INTELLIGENCE

**DOI:** 10.1093/geroni/igad104.2565

**Published:** 2023-12-21

**Authors:** Alaine Murawski, Vanessa Ramirez-Zohfeld, Allie Schierer, Ana Ramirez, Johnathan Mell, Jeanne Brett, Lee A Lindquist

**Affiliations:** Northwestern University, Chicago, Illinois, United States; Northwestern University, Chicago, Illinois, United States; Northwestern University, Chicago, Illinois, United States; New York University, New York City, New York, United States; University of Central Florida, Orlando, Florida, United States; Northwestern University Kellogg School of Management, Chicago, Illinois, United States; Northwestern University, Chicago, Illinois, United States

## Abstract

Family caregivers of people with dementia (PWD) experience conflicts as they navigate the healthcare system but do not have adequate training to resolve these disputes. We sought to develop and pilot test an artificial-intelligence negotiation training program, NegotiAge, for family caregivers. With NIH funding [R01AG068421], we convened a team of negotiation experts, geriatrician, social worker, and community-based family caregivers (Illinois/Florida/New York/California). Content matter experts created informational videos to teach negotiation skills. Family caregivers generated dialogue surrounding caregiver conflicts. Computer science experts input generated dialogue into the Interactive Arbitration Guide Online (IAGO) platform to develop avatar-based agents (e.g. sibling, older adult, physician) for caregivers to practice negotiating. Family caregivers review didactic material then access scenarios to negotiate (e.g. physician recommendations, sibling disagrees with care, PWD refusing support). Caregivers negotiate in real-time with avatars designed to act like humans, including emotional and irrational behaviors. Caregivers can use negotiation tactics until either mutual agreement occurs or time expires. Immediate feedback is generated from the responses chosen and tactics to assist with the negotiation skills training. Pilot testing was conducted with family caregivers of PWD (n=12) and showed that the negotiation and conflict resolution training program was feasible and usable for family caregivers. Subjects found the program to present real-world applicability. NegotiAge is an Artificial Intelligence-based Caregiver Negotiation Program, that is usable and feasible for family caregivers to become familiar with navigating conflicts seen in caring for PWD.

